# Supplementary material for: The optimal training intervention for improving the change of direction performance of adolescent team-sport athletes: a systematic review and network meta-analysis
Source: PeerJ. 2025 Feb 21;13:e18971. doi: 10.7717/peerj.18971 (PMC11849509; doi:10.7717/peerj.18971)
Supplement: Supplemental Information 1 [file peerj-13-18971-s001.docx]

Database: PubMed <inception to February 20, 2024>

Search strategy:

#1 Team-sports athlete* [Title/Abstract] or Young team-sports athlete [Title/Abstract] or Young team-sports player* [Title/Abstract] or Adolescent team-sports athlete* [Title/Abstract] or Adolescent team-sports player* [Title/Abstract]

#2 Anterior cruciate ligament [Title/Abstract] or Disease [Title/Abstract] or pain [Title/Abstract]

#3 #1 not #2

#4 Train*[Title/Abstract] or Exercise*[Title/Abstract] or Strength train*[Title/Abstract] or Plyometric train*[Title/Abstract] or Complex train*[Title/Abstract] or Combined train*[Title/Abstract] or Concurrent train*[Title/Abstract] or blood flow restriction train*[Title/Abstract] or balance train*[Title/Abstract] or repeated sprint train*[Title/Abstract] or Change of direction train*[Title/Abstract]

#5 #3 and #4

#6 Change of direction [MeSH] or Agility [MeSH]

#7 #5 and #6

#8 Randomized control trial or RCT

#9 #7 and #8
