# Supplementary material for: The optimal training intervention for improving the change of direction performance of adolescent team-sport athletes: a systematic review and network meta-analysis
Source: PeerJ. 2025 Feb 21;13:e18971. doi: 10.7717/peerj.18971 (PMC11849509; doi:10.7717/peerj.18971)
Supplement: Supplemental Information 2 [file peerj-13-18971-s002.docx]

| Study | Eligibility criteria and source | Random allocation | Concealed allocation | Baseline comparability | Blinding | Adequate follow-up (> 85%) | Intention-to-treat analysis | Between-group statistical comparisons | Reporting of point measures and measures of variability | Precision of results | Score |
| --- | --- | --- | --- | --- | --- | --- | --- | --- | --- | --- | --- |
| Beato 2018 | 1 | - | - | 1 | - | 1 | 1 | 1 | 1 | 1 | 7 |
| Bouteraa 2020 | 1 | 1 | - | 1 | - | 1 | 1 | 1 | 1 | 1 | 8 |
| Bourgeois 2017 | 1 | - | - | 1 | - | 1 | 1 | 1 | 1 | 1 | 7 |
| Chtara 2017 | 1 | - | - | 1 | - | 1 | 1 | 1 | 1 | 1 | 7 |
| Dos'Santos 2019 | 1 | - | - | 1 | - | 1 | 1 | 1 | - | 1 | 6 |
| Fiorilli 2020 | 1 | 1 | 1 | 1 | - | 1 | 1 | 1 | 1 | 1 | 9 |
| Gaamouri 2023 | 1 | 1 | - | 1 | - | 1 | 1 | 1 | 1 | 1 | 8 |
| Genc 2019 | 1 | 1 | - | 1 | - | 1 | 1 | 1 | 1 | 1 | 8 |
| Hammami 2016 | 1 | 1 | - | 1 | - | 1 | 1 | 1 | 1 | 1 | 8 |
| Hammami 2017 | 1 | 1 | - | 1 | - | 1 | 1 | 1 | 1 | 1 | 8 |
| Hammami 2018 | 1 | - | - | 1 | - | 1 | 1 | 1 | 1 | 1 | 7 |
| Hammami 2019a | 1 | 1 | - | 1 | - | 1 | 1 | 1 | 1 | 1 | 8 |
| Hammami 2019b | 1 | 1 | - | 1 | - | 1 | 1 | 1 | 1 | 1 | 8 |
| Hammami 2021 | 1 | 1 | - | 1 | - | 1 | 1 | 1 | 1 | 1 | 8 |
| Haghighi 2023 | 1 | 1 | 1 | 1 | - | 1 | 1 | 1 | 1 | 1 | 9 |
| Ince 2019 | 1 | 1 | - | 1 | - | 1 | 1 | 1 | 1 | 1 | 8 |
| Keller 2020 | 1 | 1 | - | 1 | - | 1 | 1 | 1 | 1 | 1 | 8 |
| Maio Alves 2010 | 1 | - | - | 1 | - | 1 | 1 | 1 | 1 | 1 | 7 |
| Makhlouf 2015 | 1 | 1 | 1 | 1 | - | 1 | 1 | 1 | 1 | 1 | 9 |
| Makhlouf 2018 | 1 | - | - | 1 | - | 1 | 1 | 1 | - | 1 | 6 |
| MATHISEN 2014 | 1 | - | - | 1 | - | 1 | 1 | 1 | - | 1 | 6 |
| Meylan 2009 | 1 | 1 | - | 1 | - | 1 | 1 | 1 | 1 | 1 | 8 |
| Michailidis 2019 | 1 | - | - | 1 | - | 1 | 1 | 1 | - | 1 | 6 |
| Negra 2016 | 1 | 1 | - | 1 | - | 1 | 1 | 1 | 1 | 1 | 8 |
| Negra 2020 | 1 | 1 | - | 1 | - | 1 | 1 | 1 | 1 | 1 | 8 |
| Otero-Esquina 2017 | 1 | - | - | 1 | - | 1 | 1 | 1 | 1 | 1 | 7 |
| Pavillon 2021 | 1 | 1 | - | 1 | - | 1 | 1 | 1 | 1 | 1 | 8 |
| Panagoulis 2020 | 1 | 1 | - | 1 | - | 1 | 1 | 1 | 1 | 1 | 8 |
| Ramírez-Campillo 2015 | 1 | - | - | 1 | - | 1 | 1 | 1 | - | 1 | 6 |
| Ramirez-Campillo 2018 | 1 | 1 | 1 | 1 | 1 | 1 | 1 | 1 | 1 | 1 | 9 |
| Ramirez-Campillo 2020 | 1 | 1 | 1 | 1 | 1 | 1 | 1 | 1 | 1 | 1 | 9 |
| Sáez De Villarreal 2015 | 1 | 1 | - | 1 | - | 1 | 1 | 1 | 1 | 1 | 8 |
| Sanchez-Sanchez 2019 | 1 | 1 | - | 1 | - | 1 | 1 | 1 | 1 | 1 | 8 |
| Shui Yizhou 2018 | 1 | 1 | - | 1 | - | 1 | 1 | 1 | 1 | 1 | 8 |
| Tous-Fajardo 2016 | 1 | - | - | 1 | - | 1 | 1 | 1 | 1 | 1 | 7 |
| Xu Meng 2015 | 1 | 1 | - | 1 | - | 1 | 1 | 1 | 1 | 1 | 8 |
