# Supplementary material for: The optimal training intervention for improving the change of direction performance of adolescent team-sport athletes: a systematic review and network meta-analysis
Source: PeerJ. 2025 Feb 21;13:e18971. doi: 10.7717/peerj.18971 (PMC11849509; doi:10.7717/peerj.18971)
Supplement: Supplemental Information 3 [file peerj-13-18971-s003.docx]

Overall COD performance analysis

| Interventions | P-score |
| --- | --- |
| EOT | 0.9775 |
| INT | 0.7521 |
| CT | 0.6306 |
| COM | 0.5542 |
| HIIT | 0.5362 |
| RST | 0.5277 |
| PT | 0.5128 |
| CODT | 0.4637 |
| TRT | 0.3989 |
| TMT | 0.0971 |
| CON | 0.0492 |

COD performance analysis with angle below 90°

| Interventions | P-score |
| --- | --- |
| COM | 0.9646 |
| PT | 0.6553 |
| TRT | 0.6411 |
| CT | 0.3131 |
| EOT | 0.2806 |
| CON | 0.1452 |

COD performance analysis with angle above 90°

| Interventions | P-score |
| --- | --- |
| EOT | 0.8843 |
| INT | 0.7616 |
| COM | 0.6032 |
| RST | 0.5789 |
| CODT | 0.5736 |
| HIIT | 0.5498 |
| TRT | 0.4515 |
| PT | 0.422 |
| TMT | 0.1108 |
| CON | 0.0643 |
