# Supplementary material for: The optimal training intervention for improving the change of direction performance of adolescent team-sport athletes: a systematic review and network meta-analysis
Source: PeerJ. 2025 Feb 21;13:e18971. doi: 10.7717/peerj.18971 (PMC11849509; doi:10.7717/peerj.18971)
Supplement: Supplemental Information 4 [file peerj-13-18971-s004.docx]

# Appendix 4. Evaluation of Heterogeneity and Inconsistency

## Appendix 4.1 Evaluation of Heterogeneity

We use the tau square (τ^2^) test and p-value to qualitatively analyze the statistical heterogeneity between the studies. The larger the τ^2^ and the smaller the p-value, the greater the possibility of heterogeneity; on the contrary, the smaller the existence heterogeneity. In addition, I^2^ is a parameter for quantitative analysis of the heterogeneity between the results of each study. It’ s value is distributed from 0-100%. When I^2^ is less than 25%, it means that the heterogeneity is low; 25%-50% means that the heterogeneity is moderate; I^2^ > 75% means high heterogeneity. In summary, when I^2^ > 50%, it means that there is substantial heterogeneity.

|  | τ^2^ | Q | df | P | I^2^ | Heterogeneity assessment |
| --- | --- | --- | --- | --- | --- | --- |
| Overall COD performance analysis | 0.3141 | 228.9 | 74 | < 0.0001 | 67.7% | moderate to high |
| COD performance analysis with angle below 90° | 0 | 2 | 3 | 0.5715 | 0% | Low |
| COD performance analysis with angle above 90° | 0.2758 | 94.95 | 37 | < 0.0001 | 61% | moderate to high |

## Appendix 4.2 Summary of the global inconsistency and SIDE-splitting results

| Outcomes | the Design-by-Treatment test | | | |
| --- | --- | --- | --- | --- |
|  | Q | df | τ^2^ | p-value |
| Overall COD performance analysis | 18.12 | 13 | 0.6008 | 0.1532 |
| COD performance analysis with angle less than 90° | 0 | 0 | 0 | -- |
| COD performance analysis with angle greater than 90° | 3.73 | 8 | 0.3467 | 0.8808 |

## Appendix 4.3 Node inconsistency test

## Appendix 4.3.1 Overall COD performance analysis

| **Comparison** | **k** | **prop** | **NMA** | **Direct** | **Indirect** | **Diff** | **z** | **p** |
| --- | --- | --- | --- | --- | --- | --- | --- | --- |
| CODT VS COM | 1 | 0.21 | -0.0305 | -0.0800 | -0.0172 | -0.0628 | -0.07 | 0.9412 |
| CODT VS CON | 4 | 0.70 | -0.8276 | -0.6807 | -1.1669 | 0.4863 | 0.70 | 0.4845 |
| CODT VS CT | 0 | 0 | 0.5121 | NA | 0.5121 | NA | NA | NA |
| CODT VS EOT | 0 | 0 | 0.5699 | NA | 0.5699 | NA | NA | NA |
| CODT VS HIIT | 0 | 0 | 0.4507 | NA | 0.4507 | NA | NA | NA |
| CODT VS INT | 0 | 0 | 0.5566 | NA | 0.5566 | NA | NA | NA |
| CODT VS PT | 1 | 0.20 | 0.0035 | -0.6525 | 0.1702 | -0.8227 | -0.96 | 0.3369 |
| CODT VS RST | 5 | 0.93 | 0.0685 | -0.0271 | 1.3420 | -1.3691 | -1.02 | 0.3092 |
| CODT VS TMT | 0 | 0 | -0.7949 | NA | -0.7949 | NA | NA | NA |
| CODT VS TRT | 0 | 0 | -0.0426 | NA | -0.0426 | NA | NA | NA |
| COM VS CON | 17 | 0.91 | -0.7971 | -0.7424 | -1.3430 | 0.6005 | 1.01 | 0.3149 |
| COM VS CT | 0 | 0 | 0.5426 | NA | 0.5426 | NA | NA | NA |
| COM VS EOT | 0 | 0 | 0.6004 | NA | 0.6004 | NA | NA | NA |
| COM VS HIIT | 0 | 0 | 0.4812 | NA | 0.4812 | NA | NA | NA |
| COM VS INT | 0 | 0 | 0.5871 | NA | 0.5871 | NA | NA | NA |
| COM VS PT | 0 | 0 | 0.0341 | NA | 0.0341 | NA | NA | NA |
| COM VS RST | 0 | 0 | 0.0990 | NA | 0.0990 | NA | NA | NA |
| COM VS TMT | 0 | 0 | -0.7643 | NA | -0.7643 | NA | NA | NA |
| COM VS TRT | 1 | 0.10 | -0.0120 | -1.0264 | 0.0992 | -1.1256 | -1.41 | 0.1582 |
| CT VS CON | 6 | 0.83 | -1.3396 | -1.3217 | -1.4282 | 0.1065 | 0.14 | 0.8871 |
| EOT VS CON | 0 | 0 | -1.3975 | NA | -1.3975 | NA | NA | NA |
| HIIT VS CON | 3 | 0.91 | -1.2783 | -1.2820 | -1.2406 | -0.0414 | -0.03 | 0.9775 |
| INT VS CON | 2 | 1.00 | -1.3842 | -1.3842 | NA | NA | NA | NA |
| PT VS CON | 17 | 0.72 | -0.8311 | -0.7479 | -1.0410 | 0.2931 | 0.84 | 0.3988 |
| RST VS CON | 1 | 0.31 | -0.8961 | -1.1042 | -0.8023 | -0.3020 | -0.33 | 0.7419 |
| TMT VS CON | 2 | 1.00 | -0.0327 | -0.0327 | NA | NA | NA | NA |
| TRT VS CON | 10 | 0.54 | -0.7850 | -1.1544 | -0.3524 | -0.8021 | -2.29 | 0.0221 |
| CT VS EOT | 0 | 0 | 0.0578 | NA | 0.0578 | NA | NA | NA |
| CT VS HIIT | 0 | 0 | -0.0614 | NA | -0.0614 | NA | NA | NA |
| CT VS INT | 0 | 0 | 0.0445 | NA | 0.0445 | NA | NA | NA |
| CT VS PT | 1 | 0.17 | -0.5085 | -0.6675 | -0.4757 | -0.1919 | -0.24 | 0.8102 |
| CT VS RST | 0 | 0 | -0.4436 | NA | -0.4436 | NA | NA | NA |
| CT VS TMT | 0 | 0 | -1.3069 | NA | -1.3069 | NA | NA | NA |
| CT VS TRT | 3 | 0.51 | -0.5546 | -0.9152 | -0.1796 | -0.7356 | -1.24 | 0.2155 |
| EOT VS HIIT | 0 | 0 | -0.1192 | NA | -0.1192 | NA | NA | NA |
| EOT VS INT | 0 | 0 | -0.0133 | NA | -0.0133 | NA | NA | NA |
| EOT VS PT | 2 | 0.58 | -0.5663 | -1.1250 | 0.2090 | -1.3340 | -1.69 | 0.0902 |
| EOT VS RST | 0 | 0 | -0.5014 | NA | -0.5014 | NA | NA | NA |
| EOT VS TMT | 0 | 0 | -1.3648 | NA | -1.3648 | NA | NA | NA |
| EOT VS TRT | 2 | 0.47 | -0.6124 | 0.0969 | -1.2371 | 1.3340 | 1.69 | 0.0902 |
| HIIT VS INT | 0 | 0 | 0.1059 | NA | 0.1059 | NA | NA | NA |
| HIIT VS PT | 1 | 0.30 | -0.4471 | -0.0318 | -0.6286 | 0.5968 | 0.63 | 0.5303 |
| HIIT VS RST | 0 | 0 | -0.3822 | NA | -0.3822 | NA | NA | NA |
| HIIT VS TMT | 0 | 0 | -1.2456 | NA | -1.2456 | NA | NA | NA |
| HIIT VS TRT | 0 | 0 | -0.4932 | NA | -0.4932 | NA | NA | NA |
| INT VS PT | 0 | 0 | -0.5531 | NA | -0.5531 | NA | NA | NA |
| INT VS RST | 0 | 0 | -0.4881 | NA | -0.4881 | NA | NA | NA |
| INT VS TMT | 0 | 0 | -1.3515 | NA | -1.3515 | NA | NA | NA |
| INT VS TRT | 0 | 0 | -0.5991 | NA | -0.5991 | NA | NA | NA |
| PT VS RST | 1 | 0.34 | 0.0650 | 0.6023 | -0.2124 | 0.8147 | 0.88 | 0.3799 |
| PT VS TMT | 0 | 0 | -0.7984 | NA | -0.7984 | NA | NA | NA |
| PT VS TRT | 12 | 0.59 | -0.0461 | -0.2892 | 0.3012 | -0.5903 | -1.70 | 0.0888 |
| RST VS TMT | 0 | 0 | -0.8634 | NA | -0.8634 | NA | NA | NA |
| RST VS TRT | 0 | 0 | -0.1111 | NA | -0.1111 | NA | NA | NA |
| TMT VS TRT | 0 | 0 | 0.7523 | NA | 0.7523 | NA | NA | NA |

## Appendix 4.3.2 COD performance analysis with angle below 90°

| **Comparison** | **k** | **prop** | **NMA** | **Direct** | **Indirect** | **Diff** | **z** | **p** |
| --- | --- | --- | --- | --- | --- | --- | --- | --- |
| CODT VS COM | 0 | 0 | 0.3057 | NA | 0.3057 | NA | NA | NA |
| CODT VS CON | 1 | 1.00 | -0.9268 | -0.9268 | NA | NA | NA | NA |
| CODT VS CT | 0 | 0 | -0.7085 | NA | -0.7085 | NA | NA | NA |
| CODT VS EOT | 0 | 0 | -1.8355 | NA | -1.8355 | NA | NA | NA |
| CODT VS PT | 0 | 0 | -0.5025 | NA | -0.5025 | NA | NA | NA |
| CODT VS TRT | 0 | 0 | -0.3640 | NA | -0.3640 | NA | NA | NA |
| COM VS CON | 2 | 1.00 | -1.2325 | -1.2325 | NA | NA | NA | NA |
| COM VS CT | 0 | 0 | -1.0142 | NA | -1.0142 | NA | NA | NA |
| COM VS EOT | 0 | 0 | -2.1412 | NA | -2.1412 | NA | NA | NA |
| COM VS PT | 0 | 0 | -0.8083 | NA | -0.8083 | NA | NA | NA |
| COM VS TRT | 1 | 1.00 | -0.6697 | -0.6697 | NA | NA | NA | NA |
| CT VS CON | 2 | 1.00 | -0.2183 | -0.2183 | NA | NA | NA | NA |
| EOT VS CON | 0 | 0 | -0.9087 | NA | -0.9087 | NA | NA | NA |
| PT VS CON | 2 | 1.00 | -0.4243 | -0.4243 | NA | NA | NA | NA |
| TRT VS CON | 0 | 0 | -0.5628 | NA | -0.5628 | NA | NA | NA |
| CT VS EOT | 0 | 0 | -1.1270 | NA | -1.1270 | NA | NA | NA |
| CT VS PT | 0 | 0 | 0.2060 | NA | 0.2060 | NA | NA | NA |
| CT VS TRT | 0 | 0 | 0.3445 | NA | 0.3445 | NA | NA | NA |
| EOT VS PT | 0 | 0 | 1.3329 | NA | 1.3329 | NA | NA | NA |
| EOT VS TRT | 1 | 1.00 | 1.4715 | 1.4715 | NA | NA | NA | NA |
| PT VS TRT | 0 | 0 | 0.1386 | NA | 0.1386 | NA | NA | NA |

## Appendix 4.3.3 COD performance analysis with angle above 90°

| **Comparison** | **k** | **prop** | **NMA** | **Direct** | **Indirect** | **Diff** | **z** | **p** |
| --- | --- | --- | --- | --- | --- | --- | --- | --- |
| CODT VS COM | 1 | 0.35 | 0.2191 | -0.0800 | 0.3786 | -0.4586 | -0.54 | 0.5878 |
| CODT VS CON | 2 | 0.52 | -0.8001 | -0.3117 | -1.3302 | 1.0185 | 1.42 | 0.1550 |
| CODT VS CT | 0 | 0 | 0.8685 | NA | 0.8685 | NA | NA | NA |
| CODT VS EOT | 0 | 0 | 1.7010 | NA | 1.7010 | NA | NA | NA |
| CODT VS HIIT | 0 | 0 | 0.1831 | NA | 0.1831 | NA | NA | NA |
| CODT VS INT | 0 | 0 | 0.9087 | NA | 0.9087 | NA | NA | NA |
| CODT VS PT | 1 | 0.29 | 0.0061 | -0.6516 | 0.2757 | -0.9273 | -1.12 | 0.2640 |
| CODT VS RST | 0 | 0 | 0.2608 | NA | 0.2608 | NA | NA | NA |
| CODT VS TMT | 0 | 0 | -0.7674 | NA | -0.7674 | NA | NA | NA |
| CODT VS TRT | 0 | 0 | 0.0636 | NA | 0.0636 | NA | NA | NA |
| COM VS CON | 6 | 0.89 | -1.0192 | -1.0719 | -0.6132 | -0.4586 | -0.54 | 0.5878 |
| COM VS CT | 0 | 0 | 0.6494 | NA | 0.6494 | NA | NA | NA |
| COM VS EOT | 0 | 0 | 1.4819 | NA | 1.4819 | NA | NA | NA |
| COM VS HIIT | 0 | 0 | -0.0360 | NA | -0.0360 | NA | NA | NA |
| COM VS INT | 0 | 0 | 0.6896 | NA | 0.6896 | NA | NA | NA |
| COM VS PT | 0 | 0 | -0.2130 | NA | -0.2130 | NA | NA | NA |
| COM VS RST | 0 | 0 | 0.0418 | NA | 0.0418 | NA | NA | NA |
| COM VS TMT | 0 | 0 | -0.9865 | NA | -0.9865 | NA | NA | NA |
| COM VS TRT | 0 | 0 | -0.1555 | NA | -0.1555 | NA | NA | NA |
| CT VS CON | 4 | 0.78 | -1.6686 | -1.6018 | -1.9107 | 0.3089 | 0.42 | 0.6721 |
| EOT VS CON | 0 | 0 | -2.5011 | NA | -2.5011 | NA | NA | NA |
| HIIT VS CON | 2 | 0.87 | -0.9833 | -0.9511 | -1.2085 | 0.2575 | 0.19 | 0.8523 |
| INT VS CON | 2 | 1.00 | -1.7089 | -1.7089 | NA | NA | NA | NA |
| PT VS CON | 14 | 0.88 | -0.8063 | -0.7951 | -0.8850 | 0.0899 | 0.17 | 0.8620 |
| RST VS CON | 1 | 1.00 | -1.0610 | -1.0610 | NA | NA | NA | NA |
| TMT VS CON | 2 | 1.00 | -0.0327 | -0.0327 | NA | NA | NA | NA |
| TRT VS CON | 10 | 0.90 | -0.8637 | -0.9221 | -0.3490 | -0.5731 | -0.87 | 0.3861 |
| CT VS EOT | 0 | 0 | 0.8325 | NA | 0.8325 | NA | NA | NA |
| CT VS HIIT | 0 | 0 | -0.6853 | NA | -0.6853 | NA | NA | NA |
| CT VS INT | 0 | 0 | 0.0403 | NA | 0.0403 | NA | NA | NA |
| CT VS PT | 1 | 0.26 | -0.8623 | -0.6675 | -0.9295 | 0.2619 | 0.35 | 0.7290 |
| CT VS RST | 0 | 0 | -0.6076 | NA | -0.6076 | NA | NA | NA |
| CT VS TMT | 0 | 0 | -1.6359 | NA | -1.6359 | NA | NA | NA |
| CT VS TRT | 3 | 0.69 | -0.8049 | -0.9550 | -0.4630 | -0.4920 | -0.73 | 0.4652 |
| EOT VS HIIT | 0 | 0 | -1.5179 | NA | -1.5179 | NA | NA | NA |
| EOT VS INT | 0 | 0 | -0.7923 | NA | -0.7923 | NA | NA | NA |
| EOT VS PT | 0 | 0 | -1.6948 | NA | -1.6948 | NA | NA | NA |
| EOT VS RST | 0 | 0 | -1.4401 | NA | -1.4401 | NA | NA | NA |
| EOT VS TMT | 0 | 0 | -2.4684 | NA | -2.4684 | NA | NA | NA |
| EOT VS TRT | 1 | 1.00 | -1.6374 | -1.6374 | NA | NA | NA | NA |
| HIIT VS INT | 0 | 0 | 0.7256 | NA | 0.7256 | NA | NA | NA |
| HIIT VS PT | 1 | 0.42 | -0.1770 | -0.0318 | -0.2838 | 0.2520 | 0.26 | 0.7919 |
| HIIT VS RST | 0 | 0 | 0.0777 | NA | 0.0777 | NA | NA | NA |
| HIIT VS TMT | 0 | 0 | -0.9505 | NA | -0.9505 | NA | NA | NA |
| HIIT VS TRT | 0 | 0 | -0.1195 | NA | -0.1195 | NA | NA | NA |
| INT VS PT | 0 | 0 | -0.9026 | NA | -0.9026 | NA | NA | NA |
| INT VS RST | 0 | 0 | -0.6479 | NA | 0.6479 | NA | NA | NA |
| INT VS TMT | 0 | 0 | -1.6762 | NA | -1.6762 | NA | NA | NA |
| INT VS TRT | 0 | 0 | -0.8452 | NA | -0.8452 | NA | NA | NA |
| PT VS RST | 0 | 0 | 0.2547 | NA | 0.2547 | NA | NA | NA |
| PT VS TMT | 0 | 0 | 0.7736 | NA | 0.7736 | NA | NA | NA |
| PT VS TRT | 1 | 0.13 | 0.0574 | -0.4188 | 0.1288 | -0.5476 | -0.74 | 0.4618 |
| RST VS TMT | 0 | 0 | -1.0283 | NA | -1.0283 | NA | NA | NA |
| RST VS TRT | 0 | 0 | -0.1973 | NA | -0.1973 | NA | NA | NA |
| TMT VS TRT | 0 | 0 | 0.8310 | NA | 0.8310 | NA | NA | NA |

*NA* not available, *k* Number of studies providing direct evidence, *prop* Direct evidence proportion, *nma* Estimated treatment effect (SMD) in network meta-analysis, *direct* Estimated treatment effect (SMD) derived from direct evidence, *indir.* Estimated treatment effect (SMD) derived from indirect evidence, *Diff* Difference between direct and indirect treatment estimates, *z* z-value of test for disagreement (direct versus indirect), *p* p-value of test for disagreement (direct versus indirect)
