# Supplementary material for: The optimal training intervention for improving the change of direction performance of adolescent team-sport athletes: a systematic review and network meta-analysis
Source: PeerJ. 2025 Feb 21;13:e18971. doi: 10.7717/peerj.18971 (PMC11849509; doi:10.7717/peerj.18971)
Supplement: Supplemental Information 7 [file peerj-13-18971-s007.docx]

1. The rationale for conducting the systematic review / meta-analysis;

Improving change of direction (COD) performance is crucial for enhancing the overall performance and preventing common injuries, such as ACL injuries, in adolescent team athletes. However, due to the multitude of training methods, the optimal approach to enhancing COD performance in adolescent team athletes remains unclear. This ambiguity may contribute to suboptimal competitive performance or increased susceptibility to sports injuries. Therefore, this study aims to quantitatively assess the effectiveness of different training methods in improving COD performance among adolescent team athletes through a systematic meta-analysis. The findings aim to provide coaches and fitness trainers with guidance in selecting effective training methods for enhancing COD performance in this population.

2. The contribution that it makes to knowledge in light of previously published related reports, including other meta-analyses and systematic reviews.

The ability to change direction is very important in team competitions, and athletes often need to change direction to rely on their opponents' defense and create scoring opportunities. Numerous studies have explored the impact of different training methods on the directional ability of young team athletes, but there is still inconsistency among the studies, which can affect coaches and fitness instructors in choosing appropriate training methods.
